# Prognostic efficacy of the *RTN1* gene in patients with diffuse large B-cell lymphoma

**DOI:** 10.1038/s41598-021-00746-0

**Published:** 2021-10-26

**Authors:** Mohamad Zamani-Ahmadmahmudi, Seyed Mahdi Nassiri, Amir Asadabadi

**Affiliations:** 1 Department of Clinical Science, Faculty of Veterinary Medicine, Shahid Bahonar University of Kerman, P.O Box 76169133, Kerman, Iran; 2 Department of Clinical Pathology, Faculty of Veterinary Medicine, University of Tehran, Tehran, Iran

**Keywords:** Cancer, Haematological cancer

## Abstract

Gene expression profiling has been vastly used to extract the genes that can predict the clinical outcome in patients with diverse cancers, including diffuse large B-cell lymphoma (DLBCL). With the aid of bioinformatics and computational analysis on gene expression data, various prognostic gene signatures for DLBCL have been recently developed. The major drawback of the previous signatures is their inability to correctly predict survival in external data sets. In other words, they are not reproducible in other datasets. Hence, in this study, we sought to determine the gene(s) that can reproducibly and robustly predict survival in patients with DLBCL. Gene expression data were extracted from 7 datasets containing 1636 patients (GSE10846 [n = 420], GSE31312 [n = 470], GSE11318 [n = 203], GSE32918 [n = 172], GSE4475 [n = 123], GSE69051 [n = 157], and GSE34171 [n = 91]). Genes significantly associated with overall survival were detected using the univariate Cox proportional hazards analysis with a *P* value < 0.001 and a false discovery rate (FDR) < 5%. Thereafter, significant genes common between all the datasets were extracted. Additionally, chromosomal aberrations in the corresponding region of the final common gene(s) were evaluated as copy number alterations using the single nucleotide polymorphism (SNP) data of 570 patients with DLBCL (GSE58718 [n = 242], GSE57277 [n = 148], and GSE34171 [n = 180]). Our results indicated that *reticulon family gene 1 (RTN1*) was the only gene that met our rigorous pipeline criteria and associated with a favorable clinical outcome in all the datasets (*P* < 0.001, FDR < 5%). In the multivariate Cox proportional hazards analysis, this gene remained independent of the routine international prognostic index components (i.e., age, stage, lactate dehydrogenase level, Eastern Cooperative Oncology Group [ECOG] performance status, and number of extranodal sites) (*P* < 0.0001). Furthermore, no significant chromosomal aberration was found in the *RTN1* genomic region (14q23.1: Start 59,595,976/End 59,870,966).

## Introduction

*Reticulon family gene 1* (*RTN1*) (formerly termed “neuroendocrine-specific protein” [NSP]) is a reticulon-encoding gene that is associated with the endoplasmic reticulum. Reticulons play critical roles in membrane trafficking or neuroendocrine secretion in neuroendocrine cells. *RTN1* encodes 3 variants—namely NSP-A, -B, and -C—which are attached to the endoplasmic reticulum by means of 2 putative transmembrane domains in the homologous C-terminal region^1–4^.

Previous investigations have introduced *RTN1* as a potential diagnostic/therapeutic marker of neurological diseases and cancers^2,5–7^. *RTN1* was proposed as a potential marker for carcinomas with neuroendocrine characteristics^2^. It has been shown that *RTN1* reduces the anti-apoptotic activity of a protein encoded by BCL2-like 1 (BCL2L1) (ie, B-cell lymphoma-extra large [Bcl-xL]). Indeed, *RTN1* can change the subcellular localization of the Bcl-xL protein from the mitochondria to the endoplasmic reticulum, which disrupts its anti-apoptotic action^5^.

Because of the major shortages of previous prognostic gene signatures developed based on gene expression profiling^8–13^, we sought to find the gene(s) that can reproducibly predict the clinical outcome in patients with diffuse large B-cell lymphoma (DLBCL). Some of the shortcomings of the previous signatures hindering their clinical utility include the infeasibility to reproduce a prognostic signature in external datasets, negligible overlaps between the developed signatures, and large numbers of genes in the developed prognostic genes (180 genes, 90 genes, and 27 genes in signatures developed by Lenz et al. (2008), Alizadeh et al. (2000), and Wright et al. (2003), respectively). In our efforts to find the gene(s) reproducibly associated with survival via bioinformatics and computational approaches, we obtained a surprising result: The *RTN1* gene was robustly and reliably associated with a favorable outcome in 1636 patients with DLBCL (including 7 gene expression data sets). Furthermore, the *RTN1* gene remained as one of the most powerful independent prognostic factors in comparison with the international prognostic index (IPI) components.

## Results

### *RTN1* as the most robust and reproducible prognostic gene in all the data sets

**Figure 1:**
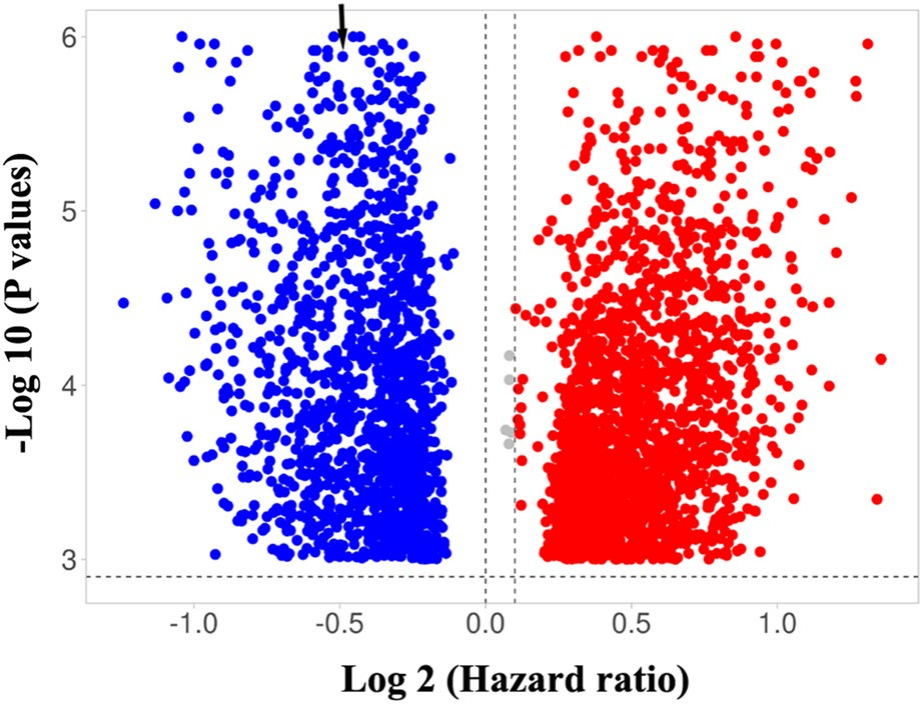
Volcano plot for GSE10846 indicating *RTN1* position (arrow) as the most reproducible prognostic gene. Similar plots for other datasets were provided in the Supplementary Figures.

**Figure 2:**
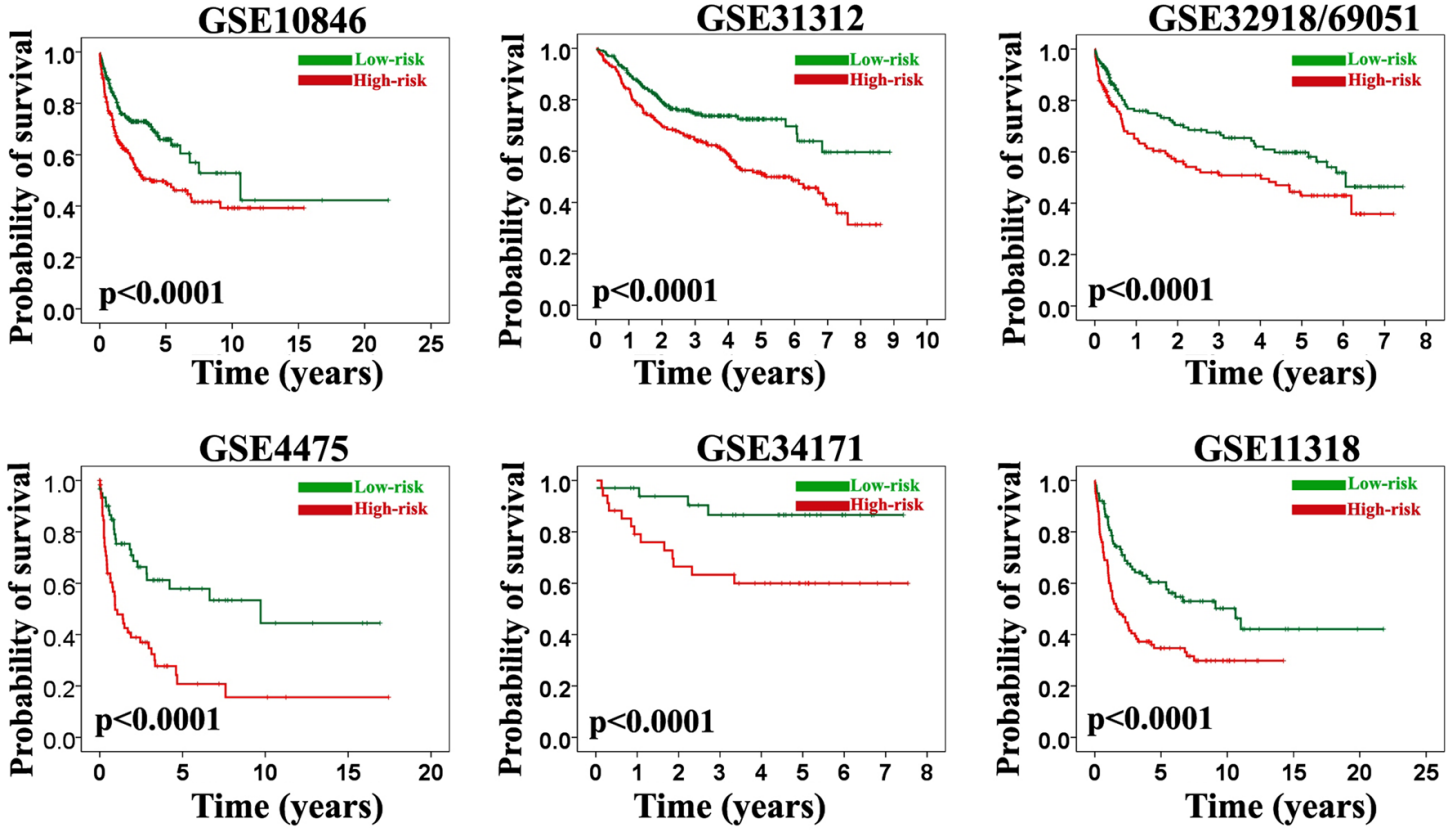
Kaplan–Meier survival analysis of the *RTN1* in various gene expression datasets. This gene was found to be significantly associated with the overall survival at a *P* value < 0.0001 in all datasets.

First, a univariate Cox proportional hazards analysis was run so as to find genes significantly associated with overall survival in all the datasets. The analysis revealed that 3 genes—namely *APOC1*, *RTN1*, and *PLAU*—fulfilled the criteria and were significantly associated with the clinical outcome at an FDR < 10% and a *P* value < 0.001 (Supplementary Table 1). When the FDR cutoff value was set at 5%, only *RTN1* met our rigorous pipeline criteria and was significantly associated with survival at a *P* value < 0.001 in the 1636 patients encompassing all the 7 data sets (Fig. 1 and Supplementary Figures). Except for GSE10846, FDRs on *RTN1* were less than 5% in various datasets (GSE31312: 3%, GSE32916/69051: 4%, GSE4475: 4%, GSE34171: 4%, and GSE11318: 3%) (*P*s < 0.001). In GSE10846, FDR was less than 1% (*P* < 0.001) (Table 1). Meanwhile, this gene showed a consistent positive association with survival in all the data sets (hazard ratio range [HR]: 0.41 to 0.79) (Table 1). Overall survival was significantly different between the low-risk and high-risk groups reconstructed based on the median of the *RTN1* expression values (> median value vs. < median value) at a *P* value < 0.0001. The rates of overall survival at 5 years in the high-risk and low-risk groups in the different datasets were as follow: GSE31312 (51% [CI: 45–56%] vs. 72% [CI: 66–77%], *P* < 0.0001), GSE10846 (48% [CI: 40–55%] vs. 67% [CI: 48–85%], *P* < 0.0001), GSE32916/69051 (43% [CI: 38–49%] vs. 60% [CI: 50–70%], *P* < 0.0001), GSE4475 (18% [CI: 10–26%] vs. 58% [CI: 44–73%], *P* < 0.0001), GSE34171 (60% [CI: 47–72%] vs. 87% [CI: 76–97%], *P* < 0.0001), and GSE11318 (36% [CI: 30–45%] vs. 60% [CI: 45–70%], *P* < 0.0001) (Fig. 2). Moreover, *RTN1* was differentially expressed between the 2 classes (i.e., long survival vs. short survival) in all the datasets in the SAM analysis (*P* < 0.001).

**Table 1:** Statistics of univariate Cox proportional hazard analysis of the *RTN1* gene in the various datasets.

Dataset	HR^a^	SE	95% CI^b^	*P* value	*FDR (%)*
*GSE10846*	0.73	0.07	0.64–0.84	**0.000**	**0.03**
*GSE31312*	0.79	0.07	0.69–0.90	**0.000**	**3**
*GSE32918/69051*	0.78	0.07	0.68–0.90	**0.000**	**4**
*GSE4475*	0.41	0.27	0.24–0.69	**0.000**	**4**
*GSE34171*	0.53	0.17	0.38–0.73	**0.000**	**4**
*GSE11318*	0.69	0.10	0.57–0.83	**0.000**	**3**

Significant *P* values were bolded.

^a^Hazard ratio, ^b^Hazard ratio 95% confidence interval.

Additionally, the multivariate Cox proportional hazards analysis indicated that *RTN1* remained independent of routine IPI components in both GSE10846 (HR: 0.78 [0.67 to 0.90]) and GSE31312 (HR: 0.77 [0.66 to 0.88]) (*Ps* < 0.0001). Nonetheless, among the IPI parameters, only age remained an independent predictor in both datasets. Some other the IPI factors were only significant in 1 dataset (Table 2). Except for the molecular subtypes, there was no significant correlation between the IPI components and *RTN1* expression (*Ps* > 0.05). Cases with *RTN1* overexpression were more frequent in the GCB-like and type 3 than in the ABC-like subtype (*Ps* < 0.05) (Table 3).

**Table 2:** Multivariate analysis of the *RTN1* and common prognostic variables in DLBCL (the IPI components).

Variable	HR^a^	SE	95% CI^b^	*P* value
**GSE10846**				
*RTN1*	0.78	0.08	0.67–0.90	**0.000**
*Molecular subtype*				
GCB-like vs. type 3	0.98	0.30	0.55–1.76	0.96
ABC-like vs. type 3	1.36	0.29	0.78–2.39	0.28
Age (≥ 60 vs. < 60 years)	1.88	0.18	1.32–2.68	**0.000**
Sex (male vs. female)	1.23	0.17	0.89–1.72	0.22
Stage (III/IV vs. I/II)	1.81	0.20	1.23–2.67	**0.000**
NES^c^ (≥ 2 vs. < 2)	1.62	0.19	1.12–2.33	**0.01**
ECOG^d^ (≥ 2 vs. < 2)	1.50	0.20	1.03–2.20	**0.04**
LDH^e^	1.58	0.20	1.07–2.35	**0.02**
**GSE31312**				
*RTN1*	0.77	0.07	0.66–0.88	**0.000**
*Molecular subtype*				
GCB-like vs. type 3	0.75	0.30	0.41–1.35	0.33
ABC-like vs. type 3	1.63	0.28	0.95–2.81	0.08
Age (≥ 60 vs. < 60 years)	2.23	0.20	1.51–3.31	**0.000**
Sex (male vs. female)	1.04	0.18	0.73–1.50	0.82
Stage (III/IV vs. I/II)	1.30	0.20	0.88–1.92	0.20
NES (≥ 2 vs. < 2)	1.16	0.34	0.59–2.27	0.67
ECOG (≥ 2 vs. < 2)	2.23	0.20	1.52–3.27	**0.000**
LDH	1.12	0.03	1.06–1.18	**0.000**

The *RTN1* gene was remained as a one of the most powerful independent prognostic factor. Significant *P* values were bolded.

^**a**^Hazard ratio, ^**b**^Hazard ratio 95% confidence interval, ^**c**^No. of extranodal sites, ^**d**^ECOG performance status, ^**e**^Lactate dehydrogenase.

**Table 3:** Correlation between the IPI components and *RTN1* expression.

Component	*GSE31312*	*GSE10846*
Molecular subtype	**0.045**	**0.031**
Age	0.351	0.324
Sex	0.113	0.660
Stage	0.579	0.927
NES	0.657	0.870
ECOG	0.819	0.643
LDH	0.141	0.050

Significant *P* value was bolded.

### Expression of *RTN1* in the molecular subtypes of DLBCL

**Figure 3:**
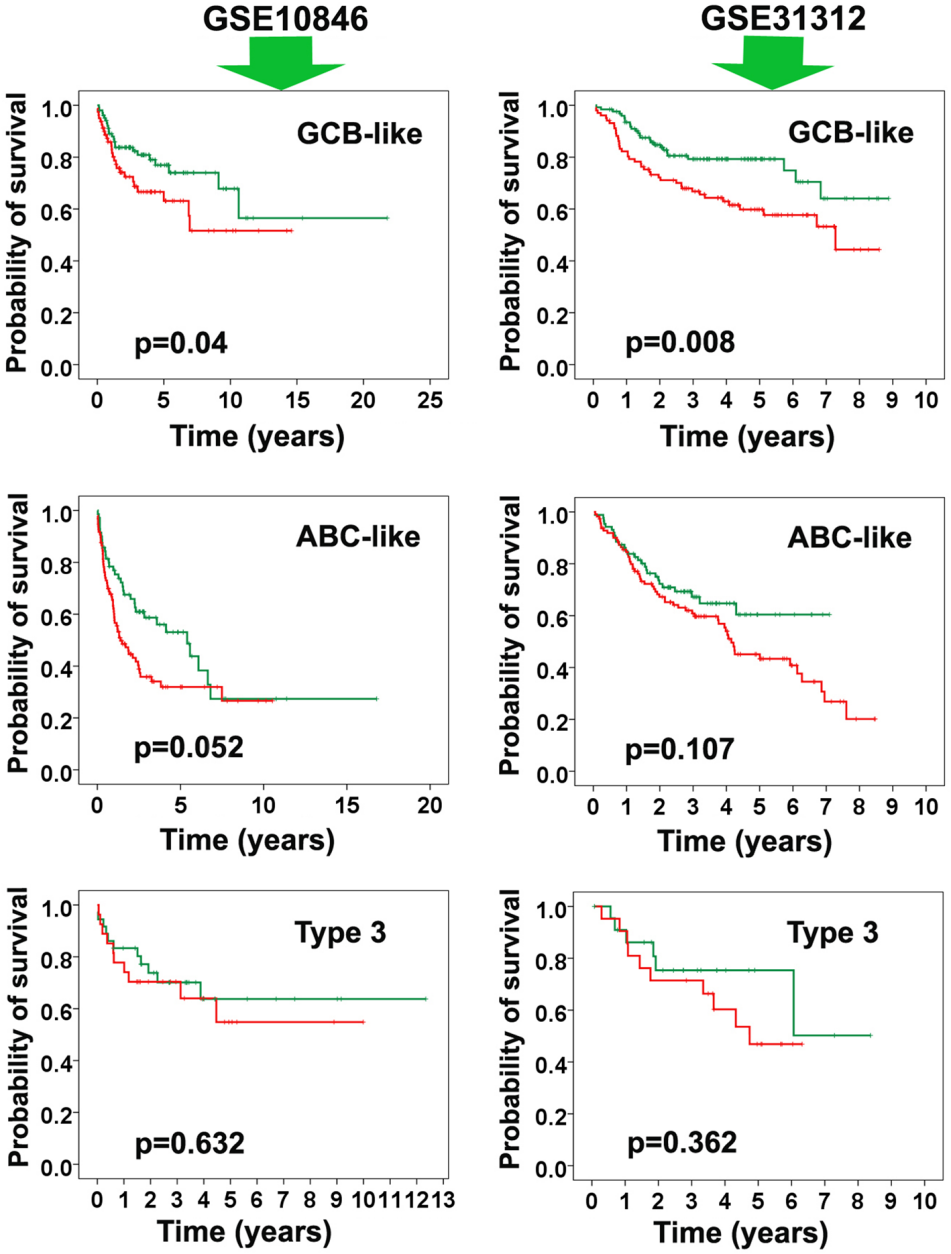
Comparison of expression of our predictor gene (*RTN1*) in three molecular subtypes of DLBCL (i.e. ABC-like, GCB-like, and type 3). Left and right panels indicate GSE10846 and GSE31312 datasets, respectively. The survival time in GCB-like was significantly different between two risk groups (*Ps* < 0.05).

The expression of *RTN1* was compared between the different molecular subtypes of DLBCL (i.e., ABC-like, GCB-like, and type 3) using the *one-way ANOVA* test. The examination indicated that the expression of *RTN1* was significantly higher in the subtype with the better overall survival (i.e., GCB-like) than in the subtype with the inferior survival (i.e., ABC-like) (*Ps* < 0.05) in both GSE10846 and GSE31312. We also checked whether overall survival was significantly different between the groups based on *RTN1* in the different molecular subtypes of DLBCL. Our analysis revealed that overall survival was significantly different between the 2 risk groups in the GCB-like subtype (*Ps* < 0.05), whereas there was no significant association between the 2 risk groups in the other subtypes (i.e., ABC-like and type 3) (Fig. 3).

### Correlation between *RTN1*, *BCL2L1*, and *MYC* expressions

**Figure 4:**
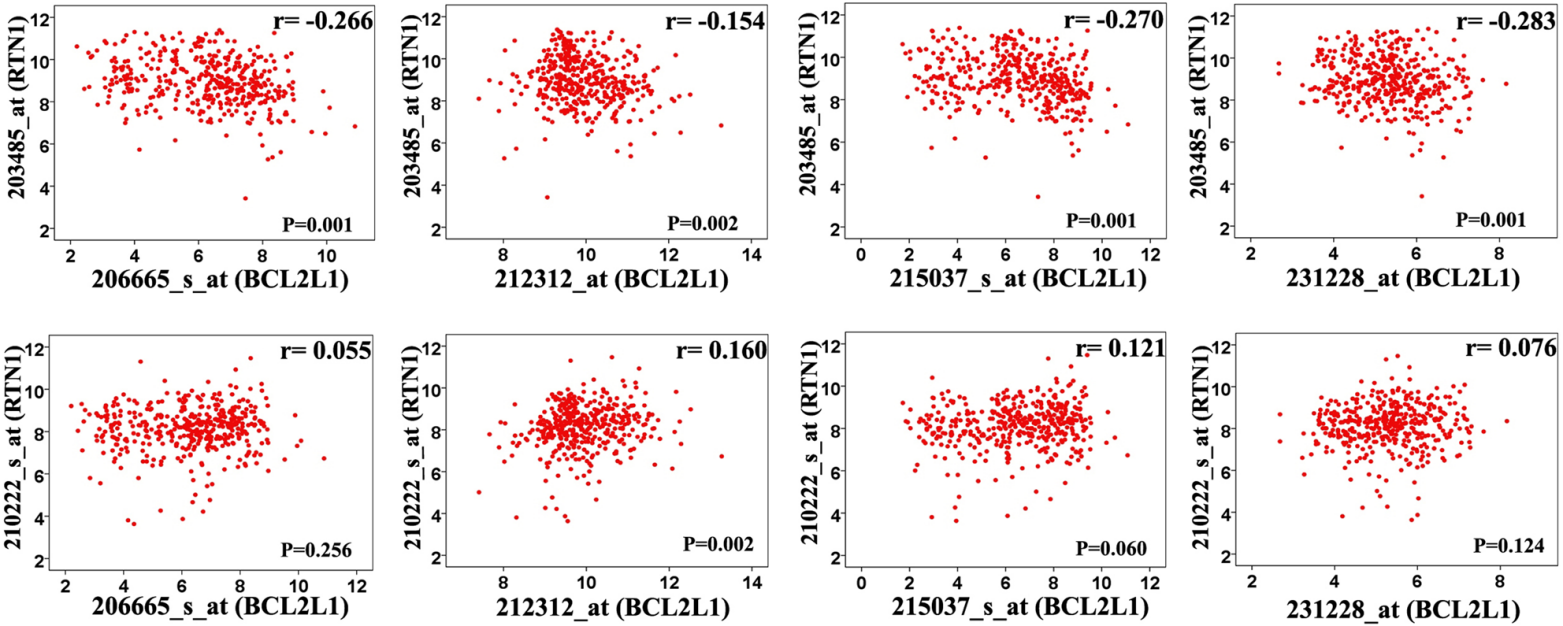
Representing scatterplot depicting the correlation between various *BCL2L1* probe-sets (x-axis) and *RTN1* probe-sets (y-axis) in GSE10846. The correlations between pairs of probes were poor and inconsistent (negative and positive correlations). Correlation analysis for other datasets was presented in Supplementary Table 2.

**Figure 5:**
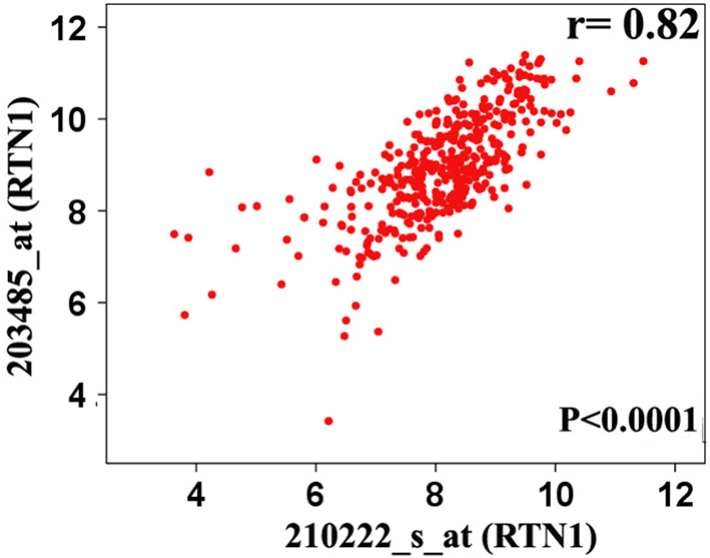
Representative scatterplot depicting the correlation between two *RTN1* probe-sets (203485_at and 210222_s_at). Correlation coefficient between two probe-sets was statistically significant (r = 0.82) (*P* < 0.05).

Tagamei et al. (2000) indicated that *RTN1* only changes the subcellular localization of Bcl-xL from mitochondria to the endoplasmic reticulum and does not alter the expression level of the corresponding gene (i.e., *BCL2L1*). Our analysis revealed no significant and consistent correlations between the *RTN1* probe-sets (n = 2) and the *BCL2L1* probe-sets (n = 4), where some inconsistent (a mix of positive and negative results) and poor correlation coefficients (*r* < 0.59) were obtained in the different analyses (Fig. 4 and Supplementary Table 2). Nevertheless, an elevation in *RTN1* expression did not suppress *BCL2L1* expression. There was a good and significant correlation between the 2 *RTN1* probe-sets (*r* = 0.82, *P* < 0.01) (Fig. 5 and Supplementary Table 2). On the other hand, these were a significant stable negative correlation between *RTN1* and *MYC* expression in analyzed datasets (*P*s < 0.05) although the correlation coefficients were not strong enough (*r* < 0.59) (Supplementary Table 3).

### Association between* RTN1* and apoptosis or cell trafficking pathways

**Figure 6:**
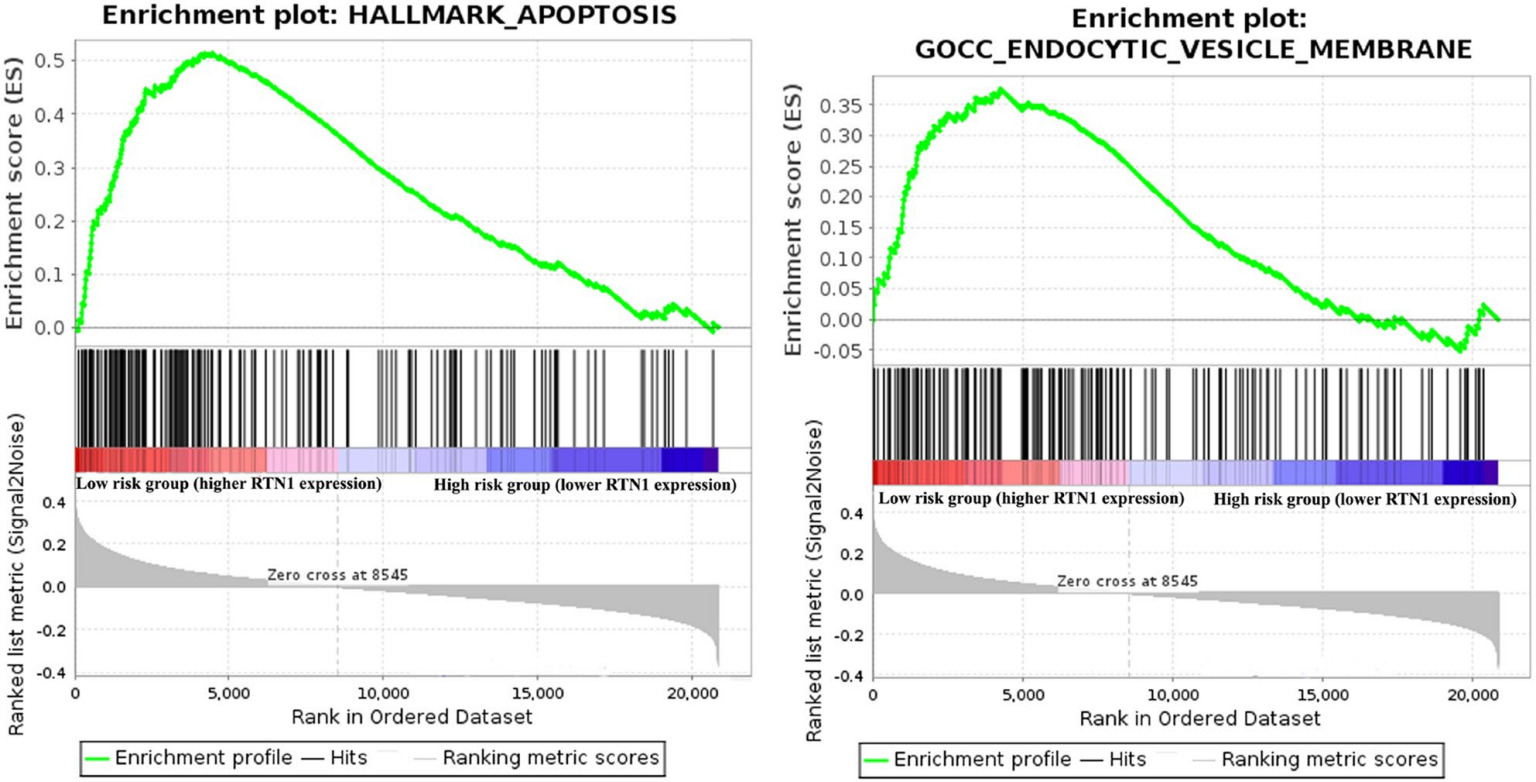
Representative gene set enrichment analysis (GSEA) indicating the enrichment of HALLMARK_APOTOSIS and GOCC_ENDOCYTIC_VESICLE_MEMBRANE gene sets in low-risk group (higher *RTN1* expression) in GSE10846 (*Ps* < 0.05). Statistics of GSEA for other datasets were provided in the Supplementary Table 4.

Our GSEA analysis revealed that the genes involved in the apoptosis pathway (i.e. HALLMARK_APOTOSIS) as well as one of the cell trafficking gene-sets (i.e. GOCC_ENDOCYTIC_VESICLE_MEMBRANE) were clearly enriched in the low-risk groups (higher *RTN1* expression) compared with the high-risk group (lower *RTN1* expression) in all datasets (*Ps* < 0.05). Furthermore, GOBP_EXOCYTIC_PROCESS pathway was also significantly enriched in the low-risk group in all datasets except GSE10846 and GSE31312 (*Ps* < 0.05) (Fig. 6 and Supplementary Table 4).

### *RTN1* at the genome level

Possible CNAs of the *RTN1* gene (14q23.1: Start 59,595,976/End 59,870,966) were checked through an analysis on the SNP data of 570 patients with DLBCL. The upstream and downstream regions that might partly include the *RTN1* region were also explored in order to detect possible aberrations. The analysis indicated no significant chromosomal aberrations in the region of the *RTN1* gene (14q23.1: Start 59,595,976/End 59,870,966). Nonetheless rare chromosomal gains (CN = 3, amplification of 59,526,724–60,901,663 segment) and losses (CN = 1, loss of 59,580,209–60,388,423 segment) were detected in 2 samples of GSE58718 and GSE34171, correspondingly, where these abnormalities were not recurrent (frequency < 1%) (Supplementary Table 5). No chromosomal instability was found in the region of interest in GSE57277.

## Discussion

Several studies have proposed various prognostic signatures comprising different numbers of genes via gene expression analysis^8–14^. Indeed, there is a minimum of overlap between these signatures and in many situations, there is no common gene in the suggested signatures. In addition, we found no genes of the previous signatures that were reproducibly associated with the clinical outcome in our data sets. Another disadvantage of these signatures is their large number of genes. Indubitably, the use of such large signatures is impractical in routine clinical practice^15,16^.

In the current study, we assessed the prognostic efficacy of *RTN1* in several large cohorts of patients with DLBCL. This gene was consistently associated with a favorable outcome in all the datasets, comprised of 1636 patients with DLBCL. The association between the *RTN1* gene expression and a favorable clinical outcome in all the datasets was significant at an FDR < 5%, which means that the probability of a false positive was extremely low. Furthermore, it remained as a one of the most powerful independent prognostic factors in comparison with the IPI components. Although *RTN1* was not the most powerful gene associated with overall survival, it was the only gene that reproducibly predicted a favorable clinical outcome. This gene was previously reported as a member of the stromal-1 signature in a 108-gene model developed by Lenz et al. (2008). Additionally, the upregulation of *RTN1* in CXCR4- DLBCL versus CXCR4 + DLBCL was indicated, where CXCR4- and CXCR4 + subtypes were associated with better and poorer overall survival, respectively^17^.

Various roles of *RTN1* in the biology of cancers have been previously investigated. As was previously described in the introduction, *RTN1* induces its antitumor activity through interaction with Bcl-xL on the endoplasmic reticulum and reduces its anti-apoptotic activity^5^. A previous investigation revealed that a member of the RTN family (ie, RTN-1C) sensitizes neuroepithelioma cells to fenretinide-induced apoptosis through interaction with glucosylceramide synthase^18^. Moreover, *RTN1*-encoded proteins have been proposed as a category criterion for human lung cancer^19^. Previous research has also demonstrated that NSP-reticulon expression is restricted to lung carcinoma cells with a neuroendocrine phenotype^6,7,19^. Another *RTN1* paralog (i.e., RTN3) has a similar antitumor activity through the enrichment of TRAIL-mediated apoptosis via the downregulation of c-FLIP and the upregulation of death receptor 5^20^.

In light of the results of the present study, *RTN1* can be considered as a potential prognostic gene capable of predicting survival in patients with DLBCL. Further studies are to be conducted to explore the prognostic efficacy of *RTN1* in depth. In this way, the prognostic efficacy of this gene should be well again compared with the regular prognostic parameters. This gene should also be experimentally validated in large cohorts of the patients with DLBCL. Because of some major limitations, we could not collect enough homogenous DLBCLs with the confirmed survival time for subsequent experimental procedure. Indeed, survival time as the most important clinical metadata was not available for many patients in our clinical settings. Because gene expression profiling using the microarray technology is carried out with various molecular chips, heterogeneous expression patterns may be resulted. In addition, high-output methods are more time-consuming and cost-effective than simpler ones. It is recommended that the prognostic efficacy of *RTN1* is evaluated in more annotated cohorts with DLBCL using subtle techniques such as PCR, quantitative real-time PCR, immunohistochemistry (IHC) to validate the current findings.

## Materials and methods

### Datasets

The Gene Expression Omnibus (GEO) (https://www.ncbi.nlm.nih.gov/geo/) database was searched to find the gene expression profiling datasets of patients with DLBCL. Only datasets containing clinical metadata (especially overall survival) (7 datasets) were retained, and the rest were excluded. Additionally, every effort was made to select expression datasets from all types of microarray chips such as Affymetrix and Illumina, if possible. The datasets were downloaded in SOFT file format and were subsequently transformed logarithmically using tools provided in *geWorkbench 2.5.1* package^21^, if necessary. More details on the clinical characteristics of the studied datasets are provided in Table 4. The datasets included GSE10846 (n = 420), GSE31312 (n = 470), GSE32918 (n = 172), GSE69051 (n = 157), GSE4475 (n = 123), GSE11318 (n = 203), and GSE34171 (n = 91). The analyzed datasets including GSE10846, GSE4475, GSE11318, and GSE69051 contained patients treated with both R-CHOP and CHOP regimens. Furthermore, GSE31312, GSE32918 and GSE34171 contained patients treated with R-CHOP regimen (Table 4). Since GSE32918 and GSE69051 have originated from a similar research study^22^ and have some common samples, they were merged as a single data set and termed “GSE32918/69051”. The number of samples for these datasets was determined after corrections were made based on the common samples (172 samples for GSE32918 and 157 samples for GSE69051). In addition, the genetic aberrations of the desired gene(s) at the genome level were evaluated by employing 3 single nucleotide polymorphism (SNP) array data sets—namely GSE58718 (n = 242), GSE57277 (n = 148), and GSE34171 (n = 180). GSE34171 contains both gene expression and SNP data (Table 4).

**Table 4:** Clinical characteristics of the microarray datasets used in our study.

Dataset	Number of patients	Chip manufacturer	Platform	Treatment	Usage in our study
GSE10846	420	Affymetrix	GPL570	R-CHOP/ CHOP	Survival analysis
GSE31312	470	Affymetrix	GPL570	R-CHOP	Survival analysis
GSE32918	172	Illumina	GPL8432	R-CHOP	Survival analysis
GSE69051	157	Illumina	GPL14951	R-CHOP/CHOP	Survival analysis
GSE4475	123	Affymetrix	GPL96	R-CHOP/CHOP	Survival analysis
GSE11318	203	Affymetrix	GPL570	R-CHOP/CHOP	Survival analysis
GSE34171	91	Affymetrix	GPL570	R-CHOP	Survival analysis
GSE58718	242	Illumina	GPL6986	NR^a^	Chromosomal aberration
GSE57277	148	Affymetrix	GPL3720	NR	Chromosomal aberration
GSE34171	180	Affymetrix	GPL6801	R-CHOP	Chromosomal aberration

^a^Not recorded.

### Identification of the common gene(s) associated with survival in the gene expression data sets

The association between gene expression and overall survival was examined using the univariate Cox proportional hazards analysis as previously described^23,24^. In this analysis, the association between a group of covariates (genes) and the response variable (overall survival) was evaluated. The univariate Cox analysis was performed using *BRB-Array tools*, developed by Richard Simon and the BRB-ArrayTools Development Team. In this analysis, the findings were strengthened by employing rigorous pipeline criteria and retaining only genes with a *P* value < 0.001 and a false discovery rate (FDR) < 5%. Subsequently, the common gene(s) significantly associated with overall survival between all the data sets was/were extracted. For this purpose, only common gene(s) with consistent associations were selected, while genes with inconsistent associations (negatively associated with overall survival in a dataset and positively associated with overall survival in another) were excluded. Moreover, the patients were categorized into 2 risk groups (high-risk vs. low-risk) based on the median of the selected common gene expression values (> median value vs. < median value), and overall survival was compared between the groups using the Kaplan–Meier analysis and log-rank test at a *P* value < 0.01. The Kaplan–Meier analysis and the log-rank test were performed in *SPSS 16.0* package (Chicago, USA). Since *RTN1* was the only gene that fulfilled the criteria and was selected as the final gene, the subsequent analyses were exclusively performed on this gene. The prognostic efficacy of *RTN1* in various datasets was visualized through depicting a volcano plot^25^, where the hazard ratios were plotted against the logarithmic values of *P* score obtained from Cox analysis.

As a confirmatory step, we checked whether *RTN1* was differentially expressed between the 2 predefined survival classes using the significance analysis of microarray (SAM) analysis. In this analysis, 2 classes (long survival [≥ 5 y] vs. short survival [< 5 y]) were created and, thereafter, the genes that were differentially expressed were detected. The SAM analysis was performed using the method added in *BRB-Array tools*. In this analysis, the FDR and the number of permutations were set at 5% and 1000, respectively.

### Prognostic efficacy of the *RTN1* gene in a multivariate model

The prognostic efficacy of *RTN1* was also evaluated in a multivariate Cox proportional-hazards regression analysis, where the *RTN1* gene expression and all the individual components of the international prognostic index (IPI) (ie, age, stage, lactate dehydrogenase level, Eastern Cooperative Oncology Group [ECOG] performance status, and number of extranodal sites)^26^ were entered as covariate variables. Additionally, the molecular subtype (ie, ABC-like, GCB-like, and type 3) and sex were incorporated as another 2 variables into the model. 3. The IPI components in two datasets (i.e. GSE31312 and GSE10846) were treated (adjusted) as ordinal variables. Hence, two or three codes were assigned to each variable (0 and 1 or 0, 1, 2). Therefore, the components were considered as follow: molecular subtypes (GCB-like, ABC-like, type 3); age (≥ 60 and < 60 years); sex (male and female); stage (III/IV and I/II); NES (≥ 2 and < 2); ECOG4 (≥ 2 vs. < 2); and LDH (0 vs. 1). The multivariate analysis was performed on the datasets with the available clinical IPI data (i.e., GSE10846 and GSE31312). This analysis was carried out using *Survival* package (http://cran.r-project.org/package=survival) and *SPSS 16.0* package (Chicago, USA).

Furthermore, the correlation between *RTN1* expression and the IPI components was evaluated using the Pearson chi-squared test. In this analysis, patients were divided into two overexpression and none-overexpression groups based on *RTN1* expression.

### Prognostic efficacy of the *RTN1* gene in molecular subtypes of DLBCL

We also checked the prognostic worth of *RTN1* in molecular subtypes of DLBCL (i.e., ABC-like, GCB-like, and type 3) using similar strategy described above. In brief, in each subtypes two risk groups constituted based on median of the *RTN1* expression values (> median value vs. < median value) and then overall survival was compared between the groups using the Kaplan–Meier analysis and log-rank test at a *P* value < 0.01.

### Correlation between *RTN1*,* BCL2L1*, and* MYC* expressions

One of the main targets of *RTN1* is *BCL2L1* via the inhibition of its anti-apoptotic activity^5^. Moreover, BCL2L1 is a member of BCL-2 protein families deregulated in lymphoma tumors, especially DLBCL^27–29^. Accordingly, the associations between 2 probe-sets of *RTN1* (i.e., 203485_at and 210222_s_at) and 4 probe-sets of *BCL2L1* (i.e., 206665_s_at, 212312_at, 215037_s_at, and 231228_at) were evaluated using correlation analysis. We also analyzed correlation between one of the most important proto-oncogenes involved in DLBCL (i.e. *c-MYC*) and *RTN1*. As well, the association analysis between *C-MYC* (202431_s_at) and *RTN1* probe-sets was carried out. The correlations were graded based on the classification proposed by Papasouliotis et al.^30^ (i.e., *r* = 0.93 to 0.100 as excellent, *r* = 0.80 to 0.92 as good, *r* = 0.59 to 0.79 as fair, and *r* < 0.59 as poor correlations). The correlation analysis was performed using *SPSS 16.0* package (Chicago, USA) in all the datasets, and a *P* value < 0.05 was considered significant.

### Association between *RTN1* and apoptosis as well as cell trafficking pathways

The associations between *RTN1* and genes involved in apoptosis and cell trafficking pathways were evaluated using the Gene-Set Enrichment Analysis (GSEA)^31^. Apoptosis pathway (gene-set) (i.e. HALLMARK_APOTOSIS) and two cell trafficking gene-sets (i.e. GOCC_ENDOCYTIC_VESICLE_MEMBRANE, GOBP_EXOCYTIC_PROCESS) were retrieved from the Molecular Signatures Database (MSigDB) (http://software.broadinstitute.org/gsea/index.jsp). Then, the expression patterns of these gene-sets were compared between the low-risk and high-risk groups reconstructed based on the median of the *RTN1* expression values (> median value vs. < median value) in different datasets. These analyzes were run on 1000 permutations.

### Evaluation of *RTN1* at the genome level in the DLBCL samples

For the assessment of the chromosomal aberrations of the *RTN1* gene, 3 datasets—namely GSE58718 (n = 242), GSE57277 (n = 148), and GSE34171 (n = 180)—were used to extract copy number variations (CNVs) from the SNP data. GSE58718 was generated based on Illumina HumanCNV370-Duov1 DNA Analysis BeadChip, while GSE57277 and GSE34171 were generated using Affymetrix Mapping 250 K SNP Arrays. In brief, *PennCNV* package^32^ was used to call and analyze the CNV data. For the Illumina datasets, signal intensity data in the form of log R ratios (LRRs) and B allele frequencies (BAFs) were directly generated from the downloaded raw file. For the Affymetrix datasets, LRRS and BAFs were calculated by processing raw intensity (.CEL) files in *Affymetrix Power Tools* (https://www.affymetrix.com/support/developer/powertools/changelog/index.html), followed by *PennCNV-Affy* package. Finally, these LRRS and BAFs were used to generate CNV calls. CNVs with lengths < 1 kb, confidence scores < 10, or containing < 5 SNPs were discarded. A CNV was considered to be a recurring acquired copy number alteration (rCNA) if it occurred in more than 2.5% of the patients and was not reported in the Database of Genomic Variants, build 36 (hg18) (DGV, http://projects.tcag.ca/variation/)^33^. The location of *RTN1* was explored on chromosome 14 (14q23.1: Start 59,595,976/End 59,870,966) for chromosomal aberrations.

### Ethical standards

Our study was performed using datasets deposited in GEO database. Hence, no ethical approval was required.

## Supplementary Information

Supplementary Figures.

Supplementary Tables.

## Data Availability

The datasets in the manuscript were deposited in GEO database (http://www.ncbi.nlm.nih.gov/geo/) with the accession number GSE10846, GSE31312, GSE32918, GSE69051, GSE4475, GSE11318, GSE34171, GSE58718, GSE57277, and GSE34171. Other supporting data are included as supplementary files.
