# Supplementary material for: Prognostic efficacy of the RTN1 gene in patients with diffuse large B-cell lymphoma
Source: Sci Rep. 2021 Oct 26;11:21098. doi: 10.1038/s41598-021-00746-0 (PMC8548397; doi:10.1038/s41598-021-00746-0)

**Supplementary Figures**. Volcano plots indicating *RTN1* position (arrows) in various datasets. The hazard ratios were plotted against the logarithmic values of *P* score (–Log 10 *P* values)


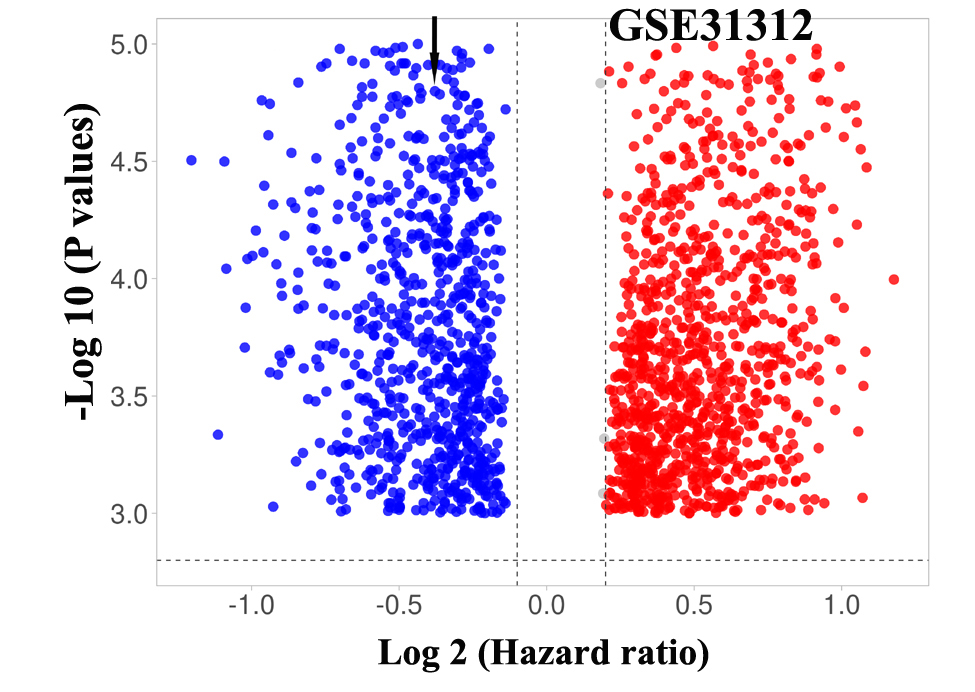


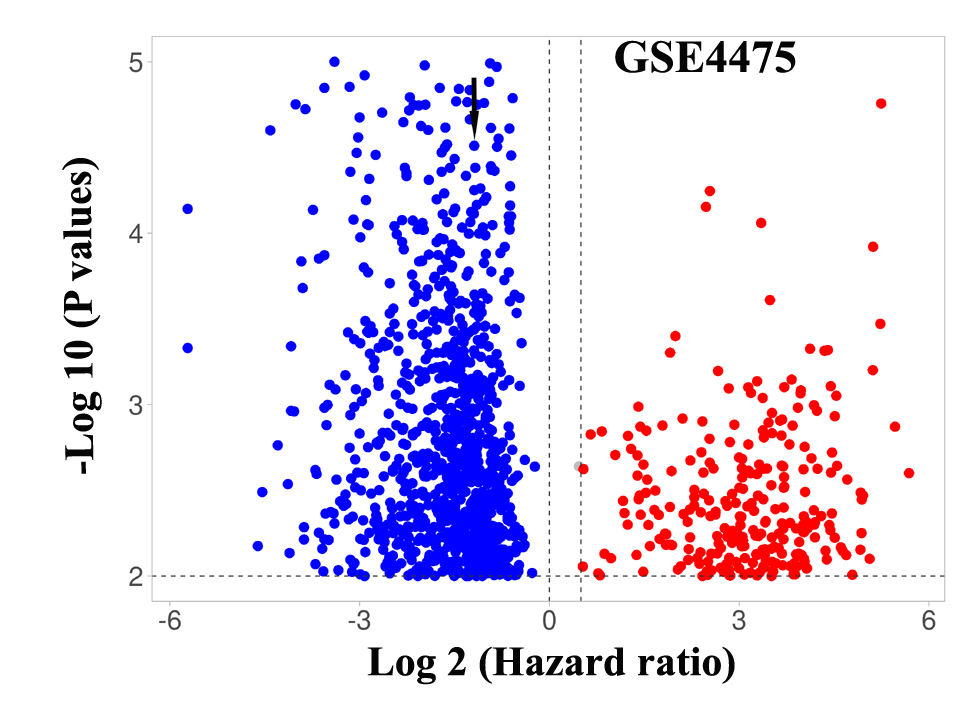


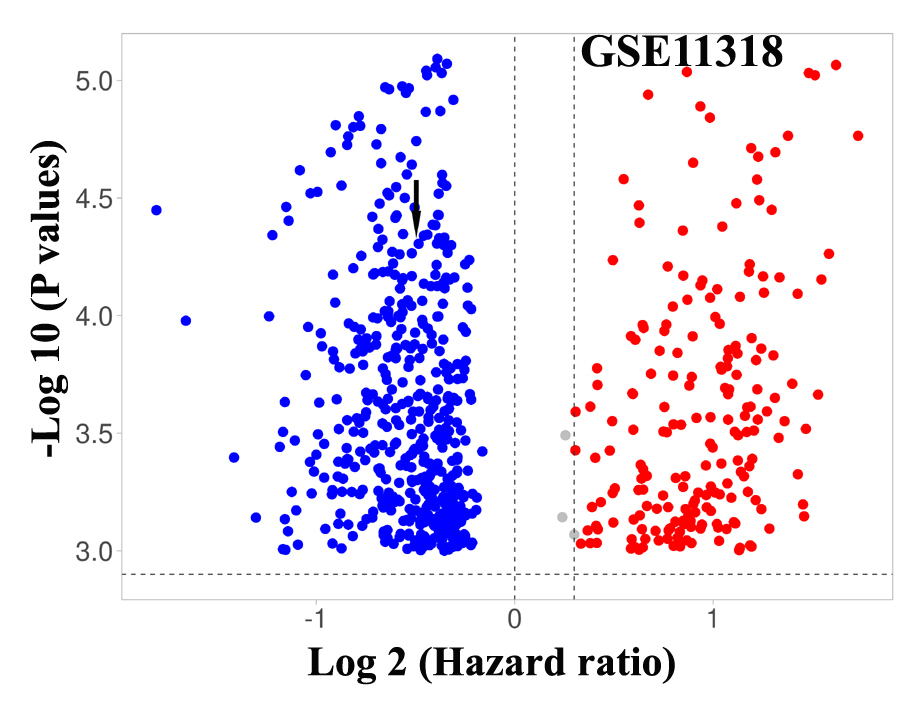


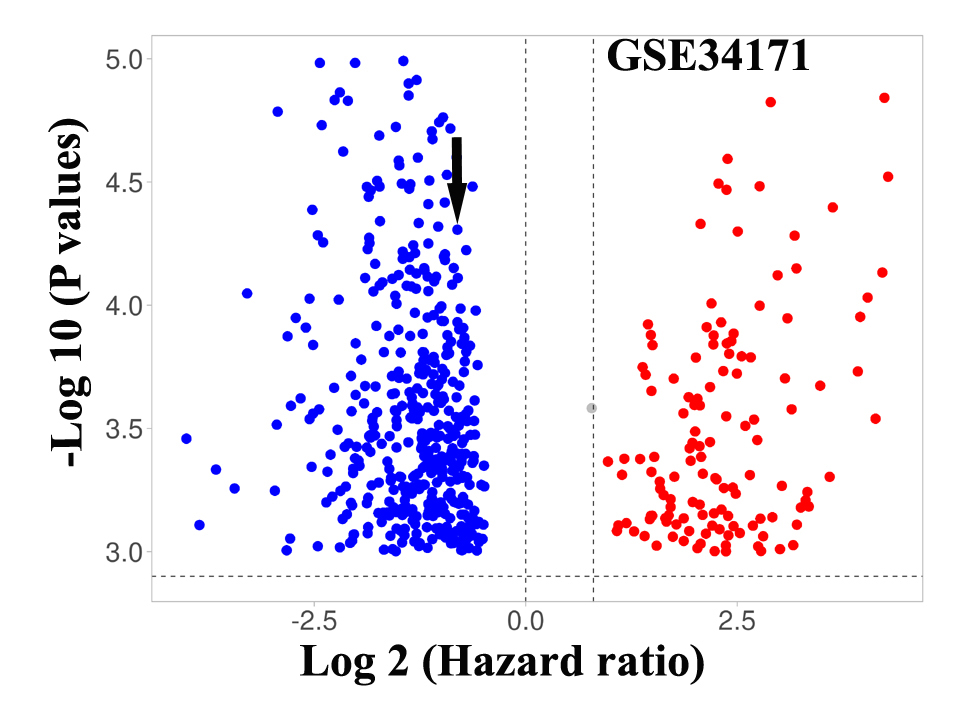


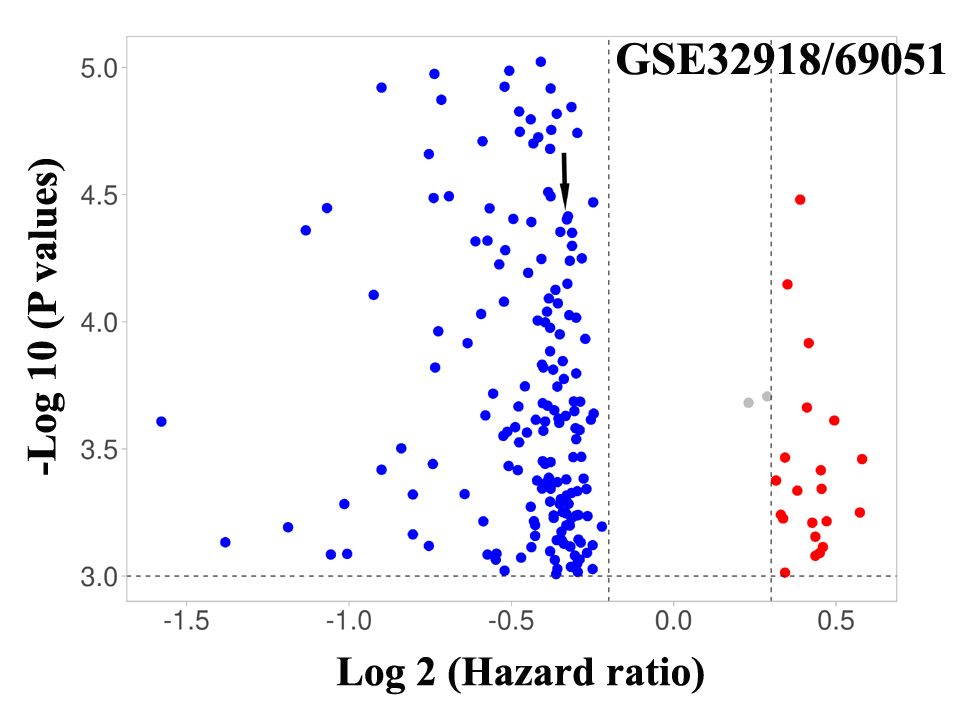

Supplement: Supplementary file 1 — Supplementary Figures. [file 41598_2021_746_MOESM1_ESM.docx]
