# Supplementary material for: Prognostic efficacy of the RTN1 gene in patients with diffuse large B-cell lymphoma
Source: Sci Rep. 2021 Oct 26;11:21098. doi: 10.1038/s41598-021-00746-0 (PMC8548397; doi:10.1038/s41598-021-00746-0)
Supplement: Supplementary file 2 — Supplementary Tables. [file 41598_2021_746_MOESM2_ESM.docx]

| Supplementary Table 1. Univariate Cox proportional hazards analysis on *APOC1*, *PLAU*, and *RTN1*. In each cell, first value indicates hazard ratio and value in parenthesis indicated FDR. | | | | | | |
| --- | --- | --- | --- | --- | --- | --- |
|  | GSE31312 | GSE10846 | GSE4475 | GSE34171 | GSE32918/69051 | GSE11318 |
| *APOC1* | 0.772 (1%) | 0.815 (9%) | 0.391 (3%) | 0.635 (9%) | 0.571 (3%) | 0.645 (3%) |
| *PLAU* | 0.769 (8%) | 0.785 (<1%) | 0.326 (1%) | 0.492 (4%) | 0.795 (9%) | 0.695 (3%) |
| *RTN1* | 0.789 (3%) | 0.731 (<1%) | 0.403 (4%) | 0.526 (4%) | 0.780 (4%) | 0.693 (3%) |

| Supplementary Table 2. The Pearson's correlation coefficient between *RTN1* and *BCL2L1* transcripts in different datasets. In each cell, the values in the parenthesis denote statistical *P-* values. | | | | | | | |
| --- | --- | --- | --- | --- | --- | --- | --- |
| *GSE31312* |  | 203485_at (RTN1) | 210222_s_at (RTN1) | 206665_s_at (BCL2L1) | 212312_at (BCL2L1) | 215037_s_at (BCL2L1) | 231228_at (BCL2L1) |
|  | 203485_at (RTN1) | - | 0.88 (<0.0001) | 0.061 (0.19) | -0.015 (0.74) | 0.092 (0.04) | -0.149 (0.001) |
|  | 210222_s_at (RTN1) | 0.88 (<0.0001) | - | 0.04 (0.39) | 0.107 (0.02) | 0.056 (0.23) | 0.08 (0.08) |
| *GSE34171* |  |  |  |  |  |  |  |
|  | 203485_at (RTN1) | - | 0.872 (<0.0001) | -0.246 (0.04) | -0.107 (0.38) | 0.12 (0.33) | 0.038 (0.76) |
|  | 210222_s_at (RTN1) | 0.872 (<0.0001) | - | -0.153 (0.21) | -0.014 (0.90) | 0.192 (0.12) | 0.073 (0.55) |
| *GSE4475* |  |  |  |  |  |  |  |
|  | 203485_at (RTN1) | - | 0.92 (<0.0001) | 0.085 (0.07) | 0.080 (0.07) | 0.256 (0.004) | -^1^ |
|  | 210222_s_at (RTN1) | 0.92 (<0.0001) | - | 0.078 (0.22) | 0. 011 (0.87) | 0.247 (0.006) | -^1^ |
| *GSE11318* |  |  |  |  |  |  |  |
|  | 203485_at (RTN1) | - | 0.86 (<0.0001) | -0.07 (0.32) | 0.081 (0.25) | -0.151 (0.03) | -0.102 (0.15) |
|  | 210222_s_at (RTN1) | 0.86 (<0.0001) | - | 0.062 (0.38) | 0.16 (0.02) | 0.009 (0.89) | -0.028 (0.69) |
| ^1^ The transcript *231228_at (BCL2L1)* wasn’t presented in the platform used by GSE4475 (GPL96). | | | | | | | |

| Supplementary Table 3. The Pearson's correlation coefficient between *RTN1* and *MYC* transcripts in different datasets. In each cell, the values in the parenthesis denote statistical *P-* values. | | | | |
| --- | --- | --- | --- | --- |
| *GSE31312* |  | 203485_at (RTN1) | 210222_s_at (RTN1) | 202431_s_at (MYC) |
|  | 203485_at (RTN1) | - | 0.88 (<0.0001) | -.130 (0.005) |
|  | 210222_s_at (RTN1) | 0.88 (<0.0001) | - | -0.260 (<0.0001) |
| *GSE10846* |  |  |  |  |
|  | 203485_at (RTN1) | - | 0.85 (<0.0001) | -0.340 (<0.0001) |
|  | 210222_s_at (RTN1) | 0.85 (<0.0001) | - | -0.275 (<0.0001) |
| *GSE34171* |  |  |  |  |
|  | 203485_at (RTN1) | - | 0.872 (<0.0001) | -0.237 (0.049) |
|  | 210222_s_at (RTN1) | 0.872 (<0.0001) | - | -0.236 (0.049) |
| *GSE4475* |  |  |  |  |
|  | 203485_at (RTN1) | - | 0.92 (<0.0001) | -0.326 (<0.0001) |
|  | 210222_s_at (RTN1) | 0.92 (<0.0001) | - | -0.406 (<0.0001) |
| *GSE11318* |  |  |  |  |
|  | 203485_at (RTN1) | - | 0.86 (<0.0001) | -0.538 (<0.0001) |
|  | 210222_s_at (RTN1) | 0.86 (<0.0001) | - | -0.402 (<0.0001) |

| Supplementary Table 4. Comparison of the expression pattern of three gene-sets related to the apoptosis and cell trafficking pathway using Gene set enrichment analysis (GSEA) between two risk groups constituted based on the median of the *RTN1* expression. Significant *P* values were bolded. | | | | | |
| --- | --- | --- | --- | --- | --- |
|  | SIZE^1^ | ES^2^ | NES^3^ | *P*-value | FDR (%) |
| *GSE10846* |  |  |  |  |  |
| HALLMARK_APOPTOSIS | 157 | 0.514 | 2.05 | **0.004** | 2.2 |
| GOCC_ENDOCYTIC_VESICLE_MEMBRANE | 156 | 0.377 | 1.55 | **0.015** | 5.3 |
| GOBP_EXOCYTIC_PROCESS | 77 | 0.215 | 0.80 | 0.771 | 82.2 |
| *GSE31312* |  |  |  |  |  |
| HALLMARK_APOPTOSIS | 157 | 0.465 | 1.90 | **0.004** | 2.5 |
| GOCC_ENDOCYTIC_VESICLE_MEMBRANE | 156 | 0.267 | 1.22 | **0.017** | 8.6 |
| GOBP_EXOCYTIC_PROCESS | 77 | 0.178 | 0.75 | 0.903 | 82.9 |
| *GSE4475* |  |  |  |  |  |
| HALLMARK_APOPTOSIS | 155 | 0.697 | 1.66 | **0.000** | 0.6 |
| GOCC_ENDOCYTIC_VESICLE_MEMBRANE | 138 | 0.654 | 1.90 | **0.000** | 0.0 |
| GOBP_EXOCYTIC_PROCESS | 59 | 0.596 | 1.90 | **0.000** | 0.0 |
| *GSE34171* |  |  |  |  |  |
| HALLMARK_APOPTOSIS | 157 | 0.501 | 1.84 | **0.006** | 1.9 |
| GOCC_ENDOCYTIC_VESICLE_MEMBRANE | 156 | 0.480 | 1.98 | **0.000** | 4.8 |
| GOBP_EXOCYTIC_PROCESS | 77 | 0.408 | 1.56 | **0.013** | 4.9 |
| *GSE32918/69051* |  |  |  |  |  |
| HALLMARK_APOPTOSIS | 153 | 0.581 | 2.06 | **0.000** | 0.0 |
| GOCC_ENDOCYTIC_VESICLE_MEMBRANE | 155 | 0.551 | 2.21 | **0.000** | 0.0 |
| GOBP_EXOCYTIC_PROCESS | 77 | 0.444 | 1.75 | **0.000** | 0.8 |
| *GSE11318* |  |  |  |  |  |
| HALLMARK_APOPTOSIS | 157 | 0.587 | 2.07 | **0.002** | 1.3 |
| GOCC_ENDOCYTIC_VESICLE_MEMBRANE | 156 | 0.532 | 2.06 | **0.000** | 0.75 |
| GOBP_EXOCYTIC_PROCESS | 77 | 0.404 | 1.47 | **0.041** | 7 |
| ^1^ Number of genes which had significant role in enrichment pattern, ^2^ Enrichment score, ^3^ Normalized enrichment score | | | | | |

| Supplementary Table 5. Chromosomal aberrations including one loss segment and one gain segment in *RTN1* gene | | | | | | | |
| --- | --- | --- | --- | --- | --- | --- | --- |
|  | Segment | Num of snp | length | State, CN | Startsnp | endsnp |  |
| chr14 | 59526724-60901663 | 683 | 1,374,940 | 5, CN=3 | SNP_A-2027127 | SNP_A-1906709 |  |
| chr14 | 59580209-60388423 | 405 | 808,215 | 2, CN=1 | CN_679901 | CN_684247 |  |
